# Molecular Mechanisms Underlying Progression and Spontaneous Regression in Desmoid Tumors

**DOI:** 10.3390/ijms27198887

**Published:** 2026-10-07

**Authors:** Shinji Miwa, Katsuhiro Hayashi, Takashi Higuchi, Hirotaka Yonezawa, Sei Morinaga, Yohei Asano, Satoru Demura

**Affiliations:** Department of Orthopedic Surgery, Graduate School of Medical Sciences, Kanazawa University, Kanazawa 920-8640, Japan

**Keywords:** desmoid tumor, spontaneous regression, mechanism, predictive factor

## Abstract

Desmoid-type fibromatosis, also known as desmoid tumor (DT), is a rare and locally aggressive soft tissue neoplasm driven by constitutive activation of the Wnt/β-catenin signaling pathway, most commonly through *CTNNB1* or *APC* alterations. While some tumors progress and cause substantial morbidity, approximately 25–38% undergo spontaneous regression during active surveillance, which is currently recommended as the first-line management strategy. Although advances in next-generation sequencing and transcriptomic profiling have elucidated the molecular drivers of tumor progression, the molecular mechanisms underlying spontaneous regression remain poorly understood. This review summarizes current knowledge of the molecular pathogenesis of DT and proposed mechanisms of spontaneous regression, including attenuation of Wnt/β-catenin signaling, hormonal modulation, alterations in the immune microenvironment, and crosstalk between Wnt signaling and other pathways such as Notch, PDGFR/c-Abl/HIF1α, and TGF-β. Based on the current knowledge of the molecular mechanisms in DT, spontaneous regression appears to reflect a multifactorial transition from an active proliferative state toward a quiescent, fibrotic phenotype rather than a single molecular event. Further studies integrating single-cell and spatial transcriptomics with immune profiling are warranted to identify therapeutic targets and stratify patients for active surveillance versus active treatment.

## 1. Introduction

Desmoid-type fibromatosis, also known as desmoid tumor (DT), is a rare, locally aggressive soft tissue tumor characterized by proliferation of monoclonal myofibroblasts and occurs in the chest wall, abdominal wall, and extremities [1]. Traumatic history, pregnancy, and genetic factors are related to the occurrence of DT. While complete surgical resection was previously recommended to control the disease, the recurrence rate of DT was reported to be 27–39% even in patients with negative surgical margins [2,3]. In contrast, approximately 25–38% of DTs show spontaneous regression [4,5,6]. Although the progression and regression of the disease are unpredictable, active surveillance is currently recommended as the first-line strategy, and surgical tumor resection is recommended only in patients with large tumors causing severe symptoms [1].

Previous studies have elucidated several molecular mechanisms of DT progression. However, no reports have directly elucidated the mechanism of DT regression, although several studies suggest hypothetical mechanisms underlying this phenomenon. In this review article, the molecular pathogenesis of DT progression and regression is discussed.

## 2. Molecular Pathogenesis of Desmoid Tumors

The Wnt/β-catenin signaling pathway plays an important role in the pathogenesis of DT. Under physiological conditions, the β-catenin levels are strictly regulated by the adenomatous polyposis coli (APC)/Axin/glycogen synthase kinase 3β (GSK-3β) complex. In the absence of Wnt stimulation, the APC/Axin/GSK-3β complex promotes the phosphorylation of β-catenin, leading to its ubiquitination and subsequent proteasomal degradation [7,8]. In contrast, Wnt ligand binding to Frizzled and low-density lipoprotein receptor-related protein 5/6 (LRP5/6) receptors inhibits the APC/Axin/GSK-3β complex, resulting in stabilization and accumulation of β-catenin. Then, β-catenin translocates into the nucleus and interacts with T-cell factor/lymphoid enhancer factor transcription factors to activate Wnt target genes involved in cell proliferation, differentiation, and tissue homeostasis [7]. In DT, this regulatory mechanism is frequently disrupted by activating mutations in the *CTNNB1* gene, which encodes β-catenin, or by inactivating mutations in *APC* [7]. Most *CTNNB1* mutations associated with DT occur in exon 3, which encodes the N-terminal region containing phosphorylation sites required for β-catenin degradation. These mutations impair β-catenin degradation and result in its constitutive stabilization and nuclear accumulation, leading to persistent activation of Wnt/β-catenin target genes [9,10]. Furthermore, β-catenin degradation is similarly disrupted by *APC* alterations in patients with familial adenomatous polyposis (FAP) [9,11]. Generally, only one of the *CTNNB1* or *APC* mutations is observed in DT, suggesting that dysregulation of the Wnt/β-catenin pathway is a common molecular event in the pathogenesis of DT [11]. Although *CTNNB1* or *APC* alterations were initially identified using conventional sequencing techniques, advances in genomic technologies have substantially expanded our understanding of the molecular landscape of DT. *CTNNB1* or *APC* alterations were identified in approximately 85% of DTs by Sanger sequencing, but more sensitive analyses revealed low-frequency *CTNNB1* mutations or *APC* loss in previously classified “wild-type” tumors, increasing the frequency to approximately 95% [8]. Next-generation sequencing has enabled the identification of additional genetic alterations that may contribute to the biological diversity of DT. Even tumors lacking detectable *CTNNB1* or *APC* alterations showed genomic alterations associated with Wnt activation. These findings support Wnt/β-catenin activation as a common molecular mechanism underlying DT initiation [8].

Ajabnoor et al. investigated the clinical characteristics, histologic patterns, and immunohistochemical and molecular findings of DTs using 33 specimens from 32 patients [9]. No association between different histologic patterns and clinical outcome was observed. All of the specimens showed a variable percentage of cells with nuclei positive for β-catenin. A next-generation sequencing analysis revealed non-*CTNNB1* mutations in two specimens, including *BCL10*, *MPL*, and *RBM10* gene mutations. These findings suggest that genetic alterations beyond *CTNNB1* may exist in a subset of DTs. To further characterize molecular mechanisms beyond DNA mutations, transcriptomic analyses have been performed. To identify additional driver genes in DT, Jeong et al. conducted a comparative transcriptome analysis using 29 DT and 14 reactive fibrosis tissue samples [10]. The transcriptome analysis identified 4267 differentially expressed genes specific to DT, and weighted gene coexpression network analysis elucidated a highly DT-specific gene module comprising 120 genes. Furthermore, subsequent analysis identified seven driver transcription factors (*ZNF536*, *IRX5*, *TWIST2*, *NKD2*, *PAX9*, *SHOX2*, and *SALL4*) within this DT-specific module that may contribute to the activation of fibroblasts in DT. Key genes from this module, including *TWIST2*, *LRRC15*, *CTHRC1*, *SHOX2*, and *SALL4*, demonstrated excellent diagnostic performance, and *TWIST2* had exceptionally high specificity and sensitivity, which surpassed those of β-catenin. On the other hand, various molecular alterations and heterogeneity of DT have been reported. De Bellis et al. investigated the molecular variety of DTs [11]. Transcriptomic profiling of 31 specimens from 20 DTs showed highly variable expression of transcriptomic prognostic signatures both in patients with and without progression of the tumor, and different outcomes were detected in different regions within the same tumor. Furthermore, multiomic analysis including DNA methylation, copy-number alterations, point mutations, and gene expression on 24 specimens from different regions of primary and recurrent tumors from three patients showed remarkable intra- and inter-tumor heterogeneity of genomic, epigenomic, and transcriptomic patterns. The transcriptomic profiles showed the highest degree of variability between primary and recurrent tumors from the same patient. These results indicate that recurrent DTs acquire multiple new molecular alterations, and that a biopsy specimen from a single site may lead to underestimation of the tumor heterogeneity and deteriorate the reliability of prognostic and predictive biomarkers. In addition to Wnt signaling, other signaling pathways interacting with β-catenin have also been implicated in DT progression. Among these, the Notch pathway has attracted considerable attention because of its biological crosstalk with Wnt signaling and its therapeutic potential. Shang et al. reported that DTs express components of the Notch signaling pathway, and that Hes1 (Hes Family BHLH Transcription Factor 1) was overexpressed compared to normal tissue [12]. The γ-secretase inhibitor nirogacestat suppresses Notch signaling activity and inhibits DT cell proliferation, migration, and invasion, most likely through cell cycle arrest. Gene array analysis suggests that Wnt1-inducible signaling pathway protein 2 regulates Notch and Wnt pathways. Gounder et al. conducted a phase 3 trial of nirogacestat in patients with DTs [13,14]. In this trial, 70 patients were assigned to receive nirogacestat and 72 to receive placebo. Nirogacestat provided a significant progression-free survival benefit over placebo (*p* < 0.001). The objective response rate was significantly higher with nirogacestat than with placebo (41% vs. 8%, *p* < 0.001). The complete response rate was 7% with nirogacestat and 0% with placebo, respectively. These findings support Notch pathway inhibition as a promising therapeutic strategy for DTs. In 2023, nirogacestat was approved by the US Food and Drug Administration (FDA) for adult patients with progressing DTs who require systemic treatment.

## 3. Predictive Factors of Regression and Progression

To identify patients with a high risk of progression and recurrence of DT, previous studies have shown several biological markers and prognostic factors. Bonvalot et al. compared clinical outcomes of initial observation or medical treatment with front-line surgery for abdominal DTs [6]. In the nonsurgery group, the 1- and 3-year incidences of switch to surgery were 14% and 15%, respectively. Of the 102 patients with initial observation, 29 showed spontaneous regression over a median follow-up period of 32 months. An initial tumor size of >7 cm was associated with a higher risk of strategy modification (*p* = 0.004). Schut et al. conducted a multicenter prospective cohort study (NTR4714) to assess tumor behavior and efficacy of active surveillance in 105 patients with DT [5]. In this study, progression-free survival was 58%, and the incidence of active treatment was 30%. During active surveillance, 32% of patients showed stable disease, 28% showed regression, and 40% showed progression. In this study, tumor size ≥5 cm (hazard ratio [HR] = 2.38) and S45F mutation (HR = 6.24) were associated with active treatment. In another study investigating T41A, S45F, and S45P mutations in the *CTNNB1* gene, S45F mutation was associated with a high risk of recurrence [15]. He et al. investigated predictors of local recurrence in 114 patients with sporadic DT who underwent surgical resection [16]. In this study, 35 patients (30.7%) had local recurrence, and the 2-, 5-, and 10-year RFS were 75.2%, 72.1%, and 67.0%, respectively. Multivariate analysis revealed that age <30 (HR = 4.96, *p* = 0.009), extra-abdominal site (HR = 4.08, *p* = 0.006), tumor size ≥8 cm (HR = 2.43, *p* = 0.021), and close or positive margin status (HR = 2.64, *p* = 0.027) were independent risk factors for local recurrence. Kohsaka et al. performed whole-exome sequencing and/or RNA sequencing to investigate molecular profiles in 64 patients with DT [17]. In this study, *CTNNB1* mutations at specific hotspots were observed in 56 patients (87.5%), and loss of copy number in chromosome 6 was observed in 14 patients (21.9%). Furthermore, a risk scoring system, which was developed by combining the expression of three genes (*IFI6*, *LGMN*, and *CKLF*), was a strong prognostic marker for recurrence-free survival. Fiore et al. evaluated a front-line conservative policy (wait-and-see or medical treatment) in 142 patients with DT [18]. Among the study patients, 83 received the wait-and-see policy, and 59 received medical treatment, mainly hormonal therapy and chemotherapy. The 5-year progression-free survival rate was 49.9% for the wait-and-see group and 58.6% for the medical treatment group (*p* = 0.320). Patients with primary and recurrent tumors showed similar results. Multivariate analysis identified no independent predictors of progression-free survival. These findings suggest that a conservative approach is thought to be a treatment option for both primary and recurrent DTs. Penel et al. conducted a prospective nationwide cohort study to investigate the prognostic value of *CTNNB1* mutations in patients with DT [19]. In the study, 516 patients were eligible for analysis. The first-line management was active surveillance in 352 (68.2%), surgical resection in 120 (23.3%), and systemic treatments in 44 (8.5%) patients. CTNNB1 molecular alterations, including p.T41A, p.S45F, or p.S45P, were observed in 435 patients (84.3%). Although p.S45F was significantly associated with male sex (*p* = 0.03), pain (*p* = 0.007), non-abdominal sites (*p* = 0.05), and large tumor size (*p* = 0.025), it was not significantly associated with event-free survival. Notably, van Broekhoven et al. conducted a multicenter retrospective study to investigate the prognostic value of mutations in the *CTNNB1* gene in aggressive fibromatosis [15]. In this study, the presence of T41A, S45F, and S45P mutations in the *CTNNB1* gene was reviewed in 101 patients. In total, the cumulative 5-year recurrence rate was 22.8%, and patients with the S45F mutation had a 5-year cumulative recurrence rate of 63.8%. Multivariate analysis revealed that young age (*p* = 0.011) and S45F mutation (*p* < 0.001) were independent risk factors for recurrence. Based on the studies, factors including tumor size and S45F mutation may be used to predict progression of DT. Further investigations are needed to select appropriate patients for active surveillance.

Although previous studies have identified tumor location, younger age, and larger tumor size as risk factors associated with local recurrence or tumor progression, the biological mechanisms underlying these clinical associations remain unclear. Tumor location is associated with local recurrence, and tumors in the extremities have better outcomes than those in the abdominal wall. This difference may be caused not only by the biological microenvironment, but also by resectability based on anatomical location. However, the mechanisms linking tumor location-specific features to the biological behavior of DTs remain poorly understood. Furthermore, the mechanisms underlying the high risk of local recurrence in younger patients remain unclear, although hormonal signaling, tissue remodeling, or the proliferative ability of tumor cells may contribute to the risk of local recurrence. Comprehensive assessment of clinical characteristics and molecular biomarkers may provide insights into the mechanisms underlying tumor progression, regression, and local recurrence of DTs [4,5,6].

## 4. Mechanisms Underlying Spontaneous Regression Factors Associated with Progression and Regression in Desmoid Tumors

Although DTs are locally aggressive neoplasms, spontaneous regression has been reported in approximately 25–38% of patients managed with active surveillance [4,5,6]. The diversity of the clinical behavior in DTs cannot be explained by conventional prognostic factors including tumor location, age, and tumor size. However, recent studies suggest the molecular characteristics of tumor cells and the microenvironment may contribute to these diverse clinical courses. In particular, alterations in Wnt/β-catenin signaling and interactions among tumor cells, stromal cells, and immune cells may influence the balance between tumor progression and regression. This unique biological behavior suggests that DTs retain the ability to enter a biologically quiescent state. Understanding these molecular and cellular mechanisms may explain the markedly diverse clinical behaviors.

**Figure 1:**
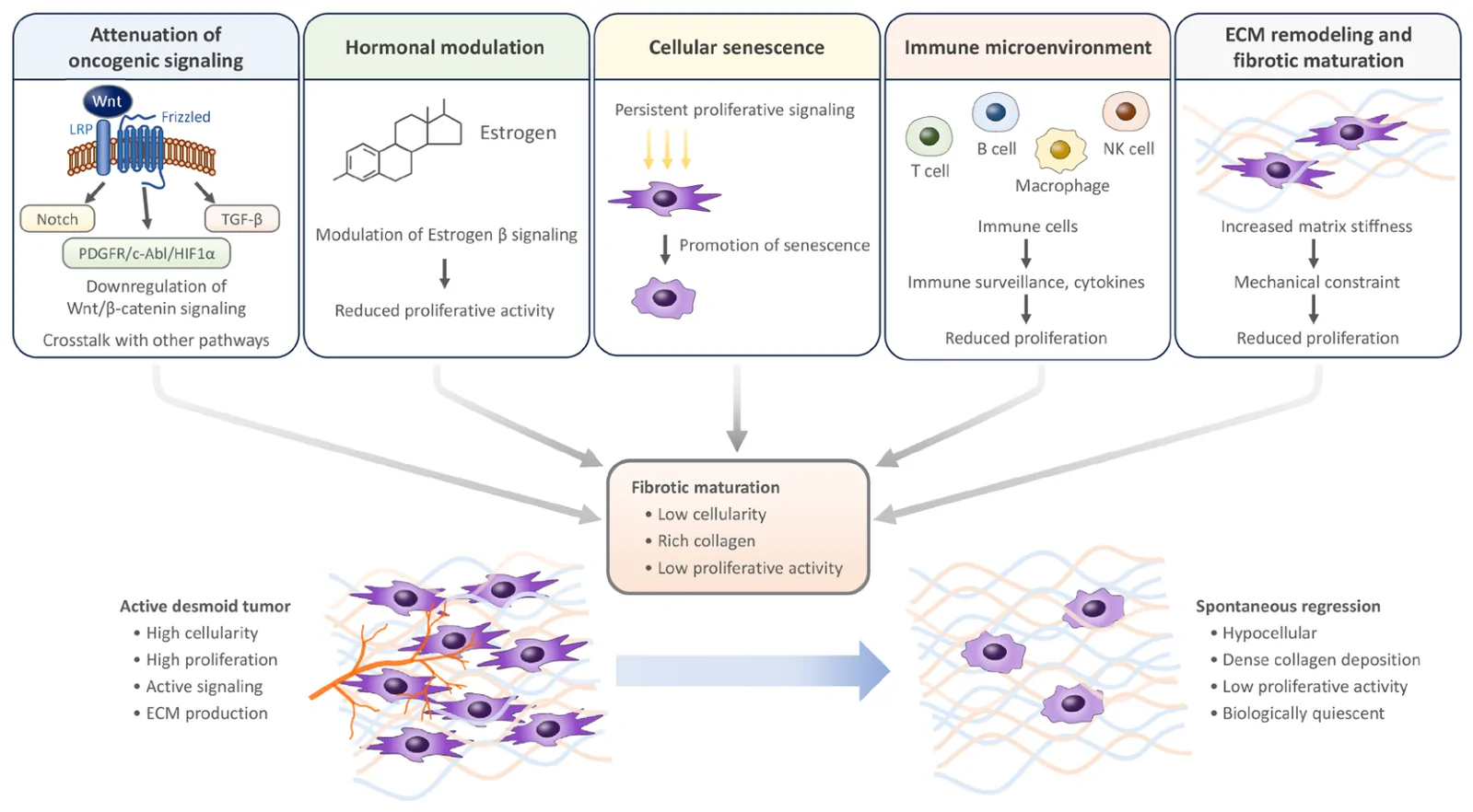
Hypothetical mechanisms of spontaneous regression in desmoid tumors.

Several mechanisms have been proposed to explain spontaneous regression, including attenuation of Wnt/β-catenin signaling, hormonal modulation, alterations in the immune microenvironment, cellular senescence, extracellular matrix remodeling and fibrotic maturation, and crosstalk between Wnt signaling and other molecular pathways (Figure 1; Table 1). Clinically, DTs exhibit a unique disease course characterized by alternating phases of progression, stabilization, and spontaneous regression. Tumor progression is associated with sustained activation of Wnt/β-catenin signaling, resulting in fibroblastic proliferation and local invasion of the tumor cells. In contrast, tumor regression is thought to be associated with reduced proliferative activity and increased fibrotic maturation, representing a transition toward a biologically quiescent phenotype. Nevertheless, the molecular mechanisms responsible for this phenotypic switch remain poorly understood.

Because constitutive activation of the Wnt/β-catenin signaling pathway, mainly through *APC* or *CTNNB1* alterations, represents the central molecular event in DT tumorigenesis [20], recent studies have focused on signaling pathways that interact with Wnt signaling and may influence tumor progression or regression. Among these, the Notch pathway has attracted considerable attention. Aberrant Wnt signaling can activate the Notch pathway, and inhibition of this crosstalk has emerged as a promising therapeutic strategy for DTs. In addition to Notch signaling, recent evidence suggests that there are interactions between Wnt signaling and multiple downstream pathways involved in hypoxia and tyrosine kinase signaling. Hu et al. demonstrated that ABL1 is a transcriptional target of β-catenin and regulates HIF1α expression and tumor proliferation in DT [21]. Knockdown of *CTNNB1* reduced HIF1α expression and hypoxia-responsive genes, whereas PDGFRβ signaling enhanced c-Abl activity and tumor cell proliferation. Sorafenib suppressed the PDGFRβ/c-Abl/HIF1α axis, reduced VEGFR2 phosphorylation, and inhibited tumor growth. Furthermore, a prognostic biomarker of PDGFRβ/c-Abl pathway activation correlated with tumor progression in placebo-treated patients, suggesting that this signaling axis may represent both a prognostic biomarker and a therapeutic target.

### 4.1. Modulation of the Wnt/β-Catenin Signaling Pathway

The Wnt/β-catenin pathway may be linked to the fibrotic maturation observed during spontaneous regression through TGF-β and connective tissue growth factor (CTGF). TGF-β and CTGF are profibrotic growth factors, downstream of the nuclear translocation of β-catenin, that lead to increased fibrogenesis. The matricellular protein CTGF, a downstream effector of TGF-β, plays a central role in tissue remodeling and fibrosis by modulating the activity of growth factors, adhesion molecules, integrins, and the extracellular matrix. In DTs, a potential mechanistic link may exist between attenuation of Wnt/β-catenin signaling and fibrotic maturation during tumor regression [22]. TGF-β signaling is also activated in DTs, as demonstrated by the frequent expression of phosphorylated SMAD2/3 [23]. Moreover, TGF-β modulates β-catenin levels in both tumoral fibroblasts and non-tumoral fibroblasts, despite the oncogenic mutations present in this gene in the tumoral fibroblasts of DTs [24]. This modulation of β-catenin levels by TGF-β may be involved in determining the tumoral phenotype of the cells. Previous reports suggest that the activity of DTs may depend not only on the CTNNB1 mutations but also on the Wnt/β-catenin signaling pathway [25]. A common feature of DTs is a dysregulated Wnt/β-catenin pathway mainly caused by gain-of-function mutations in exon 3 of the CTNNB1 gene, which result in nuclear accumulation of β-catenin [26]. In a study investigating the mechanism of DT tumorigenesis and progression, no correlation between abnormal β-catenin expression and APC gene mutation was identified [27]. On the other hand, abnormal β-catenin expression was correlated with a higher level of c-myc protein expression (*p* = 0.001), and lower apoptotic indices were correlated with positivity for c-myc (*p* = 0.007). Based on the previous studies, β-catenin has been considered as a therapeutic target in DT, and β-catenin inhibitor BC2059 was reported to have inhibitory effects on cell viability, migration, and invasion of DT cells [28]. Although β-catenin can be constitutively activated by CTNNB1 mutations, the signaling may be modulated by epigenetic regulation, tumor microenvironment, or interactions with other signaling pathways. Attenuation of β-catenin transcriptional activity may lead to a reduction in proliferation, resulting in spontaneous regression of DTs [7,8].

### 4.2. Crosstalk with Other Signaling Pathways

Although aberrant Wnt/β-catenin signaling is the central molecular driver of DTs, interactions with other signaling pathways, including Notch, PDGFR, and TGF-β, may modulate tumor behavior [20,29,30,31]. Although the association between interactions with these signaling pathways and regression of DTs is unclear, several clinical trials and basic research studies support the influence of these interactions on tumor behavior. Clinical responses to γ-secretase inhibitors and tyrosine kinase inhibitors support the biological relevance of signaling pathways as well as Wnt/β-catenin in DTs [14,32]. The PDGFRβ/c-Abl/HIF1α signaling axis may influence the biological behavior of DTs [21]. Given that activation of this pathway is associated with tumor progression and that its inhibition suppresses desmoid cell proliferation, spontaneous attenuation of this signaling cascade could represent a potential mechanism underlying spontaneous regression, although direct evidence is currently lacking.

### 4.3. Hormonal Modulation

Hormonal factors have been considered to influence the biological behavior of DTs [33,34,35,36,37]. Clinical course of DTs in patients with pregnancy, together with the historical efficacy of anti-estrogen therapy, has suggested that estrogen signaling may modulate tumor growth. Furthermore, changes in hormonal status following pregnancy or menopause have been proposed as one possible explanation for spontaneous tumor regression, although the underlying mechanisms remain poorly understood.

Leithner et al. investigated the expression of estrogen receptors α and β, progesterone receptors, HER2, Ki-67, and c-KIT in 116 tissue samples from 80 patients with DTs (46 extra-abdominal, 21 abdominal, 13 intra-abdominal) to identify potential therapeutic targets [38]. The cut-off value was 10% for estrogen receptors α and β, progesterone receptor, and c-KIT, 5% for Ki-67, and 1% for HER2. Estrogen receptor α, progesterone receptor, and HER2 were negative in all specimens (0/80), whereas estrogen receptor β was detected in only a small subset of tumors (7/80). C-KIT expression was identified in only one case (1/80). These findings suggest that classical estrogen receptor α is unlikely to be the principal mediator of hormonal effects in DTs and that alternative estrogen-related pathways may contribute to tumor pathogenesis. Subsequent studies have further supported a potential biological role for estrogen receptor β. Santti et al. investigated the expression of estrogen receptor β in 83 patients with DTs [39]. Estrogen receptor β expression varied from 0% to 74%, and estrogen receptor β was positive in 68 patients (81.9%) with a 1% cut-off value. High estrogen receptor β expression was significantly associated with Ki-67, cyclin D1, and cyclin A expression, indicating enhanced proliferative activity. Moreover, elevated estrogen receptor β expression was independently associated with a higher risk of tumor recurrence (HR 2.6; *p* = 0.02), suggesting that estrogen receptor β may serve as a prognostic biomarker rather than a direct therapeutic target.

Although several clinical trials have investigated hormonal therapy, including tamoxifen, there are insufficient data to conclude that regression rates are significantly higher than those of spontaneous regression in DTs. Hansmann et al. conducted a clinical study of tamoxifen (120 mg/day) combined with sulindac (300 mg/day) in 25 patients with DTs (17 FAP-associated, 8 sporadic) [40]. Among patients receiving this regimen as primary treatment, all sporadic DTs showed cessation of tumor growth, and 10 of 13 FAP-associated DTs achieved partial or complete regression. Based on these findings, the authors suggested that high-dose tamoxifen combined with sulindac may represent an effective primary treatment option for FAP-associated DTs. In contrast, Children’s Oncology Group conducted a phase 2 trial to evaluate the safety and efficacy of tamoxifen and sulindac (3 mg/kg, with the maximum daily dose of 300 mg for each agent) in 59 patients with DT [41]. The 2-year progression-free survival rate was not found to be significantly decreased from the natural history of untreated disease. Hormonal therapy is no longer a standard treatment for DT. In contrast, recent clinical studies of medical treatment have focused on nonsteroidal anti-inflammatory drugs, cytotoxic agents such as anthracyclines, and targeted therapies including sorafenib, nirogacestat, and varegacestat (AL102) [42].

Collectively, these findings indicate that hormonal signaling contributes to the biological behavior of DTs, although its precise role remains incompletely understood. The absence of consistent estrogen receptor α expression suggests that the therapeutic effects of anti-estrogen agents are unlikely to be mediated solely through classical estrogen signaling. Further studies are needed to clarify the contribution of estrogen receptor β and non-classical estrogen-related pathways to tumor progression and spontaneous regression.

### 4.4. Immune Microenvironment

Several studies investigated the role of the immune microenvironment and immune checkpoints in DTs. Siozopoulou et al. reported that lymphocytes organized into lymphoid aggregates at the periphery in most DTs [43]. Furthermore, a significant proportion of regulatory T cells was observed in the lymphoid aggregates. On the other hand, PD-L1 was negative on the tumor cells in most of the samples. Hashimoto et al. investigated the expression of immune molecules in 9 patients with DTs [44]. The mean expression rates of β-catenin, CD4, CD8, PD-1, PD-L1, IL-2, and IFN-γ were 43.9 ± 18.9%, 14.6 ± 6.80%, 0.75 ± 4.70%, 0 ± 0%, 5.1 ± 6.73%, 8.75 ± 6.38%, and 7.03 ± 12.1%, respectively. In another study, Hashimoto et al. reported that mean positive cell rates for β-catenin, NY-ESO-1, and MAGE-A4 were 43.9 ± 21.7, 30 ± 21.6, and 68.9 ± 20.8, respectively [45]. Based on these results, the NY-ESO-1/MAGE-A4 immune pathway may play a more important role than the PD-1/PD-L1 checkpoint pathway within the tumor microenvironment of DT.

On the other hand, Gantzer et al. performed integrated genomic, transcriptomic, and DNA methylation profiling in 76 patients with DTs [46]. In this study, mutations in canonical *CTNNB1*/*APC* genes and chromatin-remodeling genes, notably *KMT2C*, were found in more than 50% of samples. Transcriptomic analysis revealed two molecular subtypes: immune-myogenic and mesenchymal-like. The immune-myogenic subtype showed high expression of immune checkpoint genes, including *PDCD1*, *CD274*, and *CTLA4*. Furthermore, the subtype showed increased infiltration of T cells, B cells, and NK cells, higher vascularization, and elevated epithelial–mesenchymal transition plasticity and stemness. TIDE analysis predicted significantly higher ICI responsiveness in the immune-myogenic subtype.

The immune microenvironment may contribute to spontaneous regression, although evidence in DTs remains limited. Macrophages, T cells, cytokines, and inflammatory mediators, which regulate fibroblast activity and remodeling of extracellular matrix, may influence DT behavior.

### 4.5. Potential Mechanisms and Future Directions

In addition to the modulation of the Wnt/β-catenin signaling pathway, crosstalk with other signaling pathways, hormonal modulation, and the immune microenvironment, other potential mechanisms may underlie spontaneous regression of DT. DTs are characterized by myofibroblast activation and extracellular matrix production [20,29]. Persistent proliferative signaling may ultimately promote senescence in myofibroblasts [47]. This mechanism is involved in suppressing fibrosis during the normal wound-healing process and may also contribute to the spontaneous stabilization of DTs. Histologically, DTs with long period or low activity are characterized by reduced cellularity and abundant collagen deposition [48], which are consistent with maturation into inactive cells with low proliferative potential.

Extracellular matrix remodeling and fibrotic maturation have been proposed as potential mechanisms contributing to the regression of DT, although DT-specific evidence demonstrating that these processes causally drive tumor regression remains limited. DTs consist of fibroblastic cells with myofibroblastic differentiation that produce abundant collagen-rich extracellular matrix [20,29,49]. Progressive collagen deposition and extracellular matrix remodeling may mechanically constrain tumor growth and reduce cellular activity [50]. Histologically, DTs with low biological activity frequently demonstrate reduced cellularity, dense collagen deposition, and hyalinization, suggesting progressive fibrotic maturation. In a single-cell transcriptomic study comparing the transcriptional profiles of DT with those of keloid fibroblasts and normal fibroblasts, an expansion of mesenchymal fibroblasts was observed in DT [51]. Furthermore, this study revealed marked heterogeneity among fibroblast populations and suggested that COL11A1+ neural cells may play a dominant role in maintaining the profibrotic microenvironment in DT by modulating the extracellular matrix. Although the association between extracellular matrix remodeling and spontaneous regression remains unclear, these findings are consistent with the low proliferative activity observed in stable lesions. However, whether these changes are a cause or merely a consequence of spontaneous regression remains unknown. These potential mechanisms of spontaneous regression in DT remain to be investigated in basic and clinical studies.

**Table 1:** Proposed mechanisms underlying spontaneous regression in desmoid tumors. ERβ, estrogen receptor β; PDGFR, platelet-derived growth factor receptor; TGF-β, transforming growth factor β.

Proposed Mechanism	Evidence Supporting the Mechanism	Potential Contribution to Spontaneous Regression	References
Modulation of Wnt/β-catenin signaling pathway	Wnt signaling is the central driver of desmoid tumor proliferation, and pathway activity is modulated by epigenetic regulation and interacting signaling networks.	Reduced β-catenin transcriptional activity may suppress fibroblast proliferation and tumor growth.	[7,8]
Crosstalk with Notch, PDGFR/c-Abl/HIF1α, and TGF-β pathways	Notch, PDGFRβ/c-Abl/HIF1α, and TGF-β signaling interact with Wnt/β-catenin, and clinical responses to γ-secretase and tyrosine kinase inhibitors support their biological relevance.	Spontaneous attenuation of these interacting pathways could represent an additional route toward tumor quiescence, although direct evidence is lacking.	[12,14,21,32]
Hormonal modulation	Pregnancy, menopause, anti-estrogen therapy, and ERβ expression suggest hormonal influence.	Changes in estrogen signaling may reduce proliferative activity in susceptible tumors.	[38,39,40]
Cellular senescence	Persistent proliferative signaling may induce fibroblast senescence, accompanied by reduced cellularity.	Senescent fibroblasts may enter irreversible growth arrest and contribute to tumor stabilization.	[20,47]
Immune microenvironment	Immune-cell infiltration and immune-related molecular subtypes have been identified.	Immune surveillance and cytokine-mediated regulation may suppress fibroblast activity.	[43,44,46]
Extracellular matrix remodeling and fibrotic maturation	Dense collagen deposition and hyalinization characterize low-activity lesions.	Progressive fibrosis may mechanically constrain tumor growth and promote biological quiescence.	[20,50]

## 5. Conclusions

Taken together, the mechanisms of spontaneous regression in DTs seem to involve dynamic changes in oncogenic signaling, cellular senescence, regulation of the immune microenvironment, hormonal modulation, and progressive fibrotic remodeling. Spontaneous regression may reflect a transition from an active proliferative state toward a less proliferative, fibrotic, and biologically quiescent phenotype, rather than a single molecular change.

Although the precise mechanisms remain to be elucidated, accumulating evidence suggests that these biological processes underlie the heterogeneous natural course of desmoid tumors. Further studies integrating single-cell transcriptomics, spatial transcriptomics, and immune profiling may help identify predictive biomarkers of spontaneous regression and contribute to the refinement of active surveillance strategies.

## Data Availability

No new data were created or analyzed in this study. Data sharing is not applicable to this article.
